# Cytotoxicity Enhancement in Osteosarcoma with Multifunctional I-131 Radiotherapeutic Nanoparticles: In Vitro Three-Dimensional Spheroid Model and Release Kinetics Modeling

**DOI:** 10.3390/molecules29030630

**Published:** 2024-01-29

**Authors:** Suphalak Khamruang Marshall, Maneerat Taweesap, Boonyisa Saelim, Verachai Pachana, Nadeeya Benlateh, Sireetorn Sangangam, Achiraya Bumrungsin, Haswanee Kholo-asae, Issaree Wongtechanon

**Affiliations:** 1 Department of Radiology, Faculty of Medicine, Prince of Songkla University, Songkhla 90110, Thailand; 2 Molecular Imaging and Cyclotron Center, Department of Radiology, Division of Nuclear Medicine, Faculty of Medicine, Prince of Songkla University, Songkhla 90110, Thailand

**Keywords:** anticancer, chitosan, curcumin, cytotoxicity, doxorubicin, fractionated dose, I-131, nanoparticle, osteosarcoma, radiosensitivity

## Abstract

This novel radiolabeled chitosan nanoparticle, facilitated with curcumin, increased doxorubicin cytotoxicity and radiosensitivity to MG-63 osteosarcoma cells in a three-dimensional model. Delivery of the anti-epidermal growth factor receptor (EGFR) targeted carboxymethyl chitosan nanoparticles, directly labeled with Na^131^I (ICED-N), achieved deep tumor penetration in a three-dimensional model. Of three kinetic models, the Higuchi model more closely matched the experimental curve and release profiles. The anti-EGFR targeting resulted in a 513-fold greater targeting efficacy to MG-63 (EGFR+) cells than the control fibroblast (EGFR−) cells. The curcumin-enhanced ICED-N (4 × 0.925 MBq) fractionated-dose regime achieved an 18.3-fold increase in cell cytotoxicity compared to the single-dose (1 × 3.70 MBq) doxorubicin-loaded nanoparticle, and a 13.6-fold increase in cell cytotoxicity compared to the single-dose Na^131^I nanoparticle. Moreover, the ICED-N fractionated dose increased cells in the G2/M phase 8.78-fold, indicating the cell cycle arrest in the G2/M phase is associated with DNA fragmentation, and the intracellular damage is unable to be repaired. Overall, the results indicate that the fractionated dose was more efficacious than a single dose, and curcumin substantially increased doxorubicin cytotoxicity and amplified osteosarcoma cell radiosensitivity to Na^131^I.

## 1. Introduction

Curcumin is a powerful and versatile small natural molecule that has the ability to interact with various molecular targets associated with the treatment of cancer [1]. Curcumin, a natural polyphenol derived from the turmeric plant that is certified as ‘Generally Recognized As Safe’ (GRAS) by the FDA [2], has gained attention for its potential as a radiosensitizer in cancer treatment. Radiation therapy (RT) is widely acknowledged as one of the most effective non-surgical methods for managing malignant tumors; however, its efficacy is impeded by various adverse effects that affect multiple organs (including the skin), damage to healthy tissues caused by radiation, and the development of significant radioresistance in cancer cells [3,4]. Multiple processes are associated with the development of radioresistance, such as epigenetic modifications and the activation of survival signaling pathways [5]. However, research has shown that curcumin can enhance the radiosensitivity of cancer cells, making them more susceptible to the effects of ionizing radiation, and in so doing increase cytotoxicity [6]. Several studies have investigated the radiosensitizing effects of curcumin, and established curcumin enhanced the sensitivity of cells to radiation, leading to increased cell death and reduced tumor growth when combined with radiation therapy. Moreover, curcumin enhances radiation therapy by damaging the DNA in cancer cells, preventing them from growing and dividing.

Furthermore, curcumin’s anticancer properties have shown promise against osteosarcoma. Studies have investigated analogs of small curcumin molecule, which are synthetic or modified versions designed to enhance its bioactivity, stability, and solubility [7,8]. These analogs have shown they improve the effectiveness of curcumin in inhibiting osteosarcoma cell proliferation, inducing apoptosis (programmed cell death), and preventing metastasis. In addition, utilizing nanoparticles as carriers for curcumin can enhance its delivery to osteosarcoma cells. Furthermore, nanoparticles provide a targeted delivery system, improving curcumin’s bioavailability, allowing for controlled release, increasing therapeutic effectiveness, and reducing potential side effects [9,10].

One of the most significant oncogenic pathways in human cancer is the phosphatidylinositol-3-kinase (PI3K)/Akt and the mammalian target of rapamycin (mTOR) signaling pathways. Increasing evidence suggests that, in osteosarcoma, these pathways are hyperactivated and contribute to apoptosis, angiogenesis, cell cycle progression, inhibition, invasion, metastasis, tumorigenesis, and chemoresistance [11]. Akt is a protein kinase that facilitates cellular viability, while the pro-apoptotic protein Bad is a protein that induces programmed cell death, often known as apoptosis. The efficacy of curcumin-loaded nanoparticles in inducing apoptotic cell death in U2OS human osteosarcoma cells has been shown through the specific targeting of the Akt–Bad signaling pathway. The utilization of curcumin, when encapsulated inside nanoparticles, effectively directs its action towards this specific pathway through the inhibition of Akt activation and the modulation of Bad activity. Moreover, the observed increase in apoptotic cell death in U2OS human osteosarcoma cells can be attributed to the inhibition of Akt and the modification of Bad [12].

Surgical resection was the main therapy for osteosarcoma until 1970 [13,14]. The present strategy for treating newly diagnosed osteosarcoma involves administering neoadjuvant chemotherapy, followed by surgical excision of the main tumor and any clinically apparent metastatic disease. Additionally, after surgery, adjuvant chemotherapy is administered. Furthermore, after multiagent chemotherapy regimens were introduced in the 1970s, localized osteosarcoma patients’ long-term survival rates rose from 20% to 70% [15,16]. However, multidrug resistance, rapid clearance rate, non-targeted administration, and adverse effects have diminished conventional chemotherapies efficacy [17]. Anticancer, immunotherapy, gene therapy, metabolic diseases, neurological disorders, theranostics, and tissue regeneration are just a few of the areas where nanoparticle drug delivery methods have attracted considerable research interest [18,19,20,21]. Specifically, studies employing novel nanoparticle-based targeted drug delivery to combat osteosarcoma, which may effectively counteract rapid elimination and multidrug resistance, demonstrate promise [22,23,24,25]. The two most researched types of nanoparticles are liposomes and polymeric nanoparticles. Chitosan, a polymeric-based nanoparticle, has great promise for biomedical and clinical treatments; to enhance chitosan’s qualities, such as its solubility or biodegradability, a number of derivatives of chitosan have been created [26,27].

Although osteosarcoma therapy has substantially improved, the overall survival of patients with recurrence or metastasis remains low [28]. Therefore, finding new treatment targets for osteosarcoma is critical. Additionally, combination therapy involving doxorubicin and curcumin co-encapsulated in lipid-coated polymeric nanoparticles is being investigated for its potential effectiveness against human osteosarcoma [29]. This approach leverages nanoparticulate drug delivery systems to enhance the therapeutic outcomes of these drugs: (i) Doxorubicin is a potent chemotherapy drug commonly used in cancer treatment, including osteosarcoma [30,31]. It belongs to the anthracycline class of drugs and effectively inhibits DNA replication and RNA transcription within cancer cells. (ii) Curcumin is a bioactive compound found in turmeric. It has shown promise in inhibiting cancer cell growth, inducing apoptosis, and preventing tumor progression. (iii) Utilizing nanoparticles as a drug delivery system offers several advantages, including improved drug solubility, controlled release, enhanced bioavailability, and targeted drug delivery to tumor sites [32].

**Figure 1:**
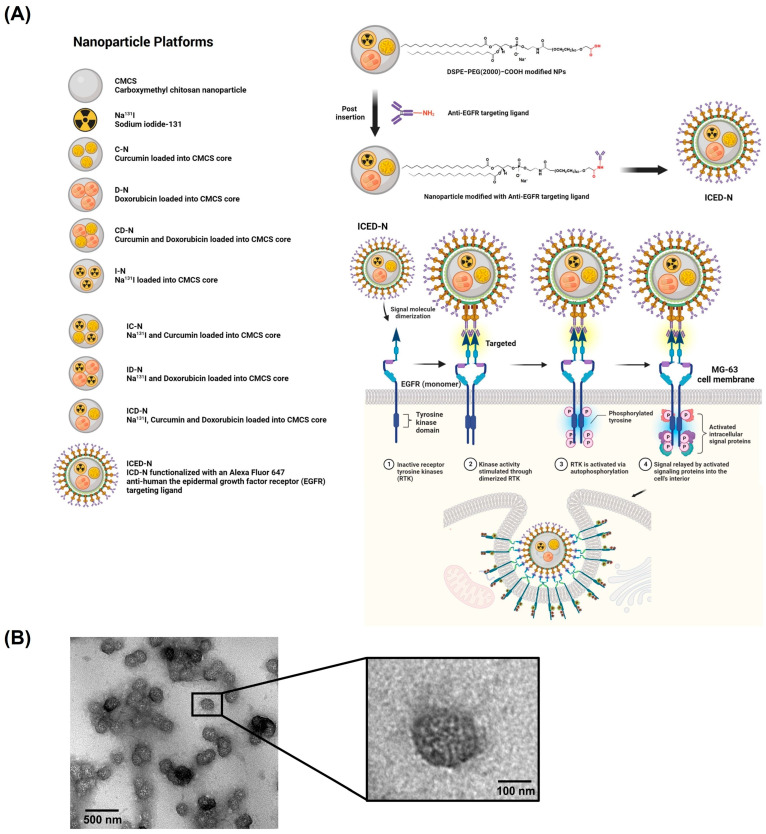
Characterization of the co-administration of doxorubicin and curcumin within Na^131^I radiolabeled carboxymethyl chitosan (CMCS) nanoparticles (ICED-N) targeted against the epidermal growth factor receptor (EGFR). (**A**) Schematic of ICED-N. (**B**) Transmission electron microscopy (TEM) image of ICED-N. Scale bar = 500 nm and 100 nm.

This study hypothesizes that the co-administration of doxorubicin and curcumin within Na^131^I radiolabeled carboxymethyl chitosan (CMCS) nanoparticles (ICED-N) targeted against the epidermal growth factor receptor (EGFR) in a three-dimensional (3D) model could potentially result in a synergistic augmentation of their respective anti-cancer properties (Figure 1A). The low attachment 3D spheroid cell culture technique is the most often used 3D strategy for generating osteosarcoma spheroids to research cell behavior and drug resistance [33]. Doxorubicin impedes the process of DNA replication, but it does not specifically target cells. Therefore, anti-epidermal growth factor receptor targeting ligands were conjugated to the ICED-N, enabling the doxorubicin to target MG-63 osteosarcoma cells selectively [34]. Conversely, curcumin has the potential to increase the anti-cancer properties of doxorubicin, alleviate its adverse effects, and additionally augment the sensitivity of cancer cells to radiation. Moreover, we show how the targeted co-encapsulation of doxorubicin and curcumin has the potential to augment the overall therapeutic effectiveness in the management of osteosarcoma while concurrently mitigating non-specific impacts and diminishing systemic toxicity.

## 2. Results

### 2.1. Physiochemical Properties and Characterization of Curcumin-Loaded Nanoparticles

To evaluate the size, polydispersity index (PDI) and zeta potential (mV) of the Na^131^I, ICED-N, ICD-N, IC-N, and C-N nanoparticles, dynamic light scattering (DLS) was carried out. Figure S1A illustrates the z-average diameter (nm) over a period of 14 days. Na^131^I ranged from ~1474 to 5882 nm, ICED-N ranged from ~229 to ~439 nm, ICD-N ranged from ~195 to 408 nm, IC-N ranged from ~95 to 247 nm, and C-N ranged from 57 to 134 nm. All experiments were carried out using CMCS nanoparticles within 24 h of fabrication. Hence, the ICED-N 258 nm average diameter was within a similar size range as Jiang et al., who studied the uptake of various chitosan nanoparticles (~250 nm diameter) in murine macrophage cells [35].

Furthermore, the polydispersity index of the Na^131^I was 0.85, and the ICED-N had a PDI of 0.22 (Figure S1B), indicating that the ICED-N has a good, narrow, uniform size [36]. Moreover, the stability of the ICED-N over 14 days is shown in Figure S1C. Figure S1B indicates the Na^131^I PDI is significantly different (*p* ≤ 0.001) compared to the ICED-N, which remained relatively stable over the 14 days. Similarly, the Na^131^I zeta potential was significantly different (*p* ≤ 0.001) compared to the ICED-N zeta potential.

Additionally, the ICED-N zeta potential was approximately −12 mV, and C-N was approximately −10 mV (Figure S1D). The presence of COO− groups’ accounts for the ICED-N negative surface charge, and is a result of the hydrogen bonding between the carboxyl group of the CMCS nanoparticle and the curcumin hydroxyl group. The ICED-N negative surface charge prevents electric repulsion that can initiate nanoparticle aggregation and indicates it has good particle stability [37]. Furthermore, the ICED-N spherical shape, size, and distribution morphology were further verified by a Transmission Electron Microscopy (TEM) image, indicating nanoparticles having a smooth and uniform surface (Figure 1B).

### 2.2. Radioactive Properties of Curcumin-Loaded Nanoparticles

**Figure 2:**
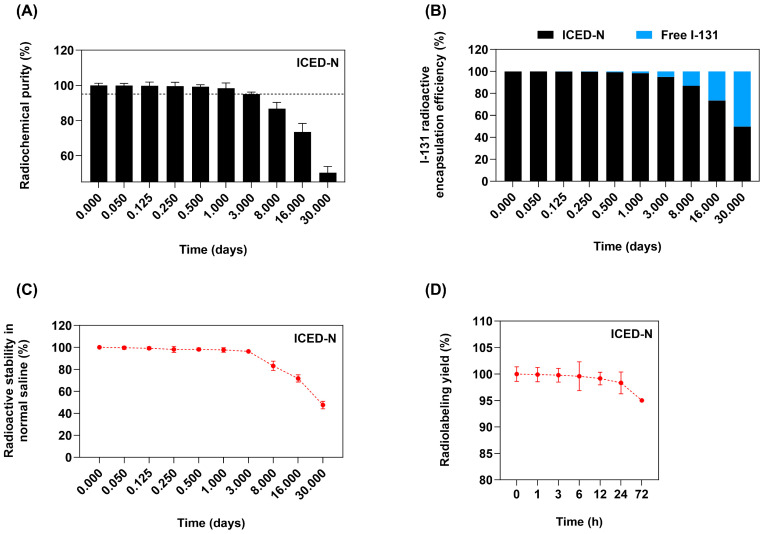
Radioactive properties of the co-administration of doxorubicin and curcumin within Na^131^I radiolabeled carboxymethyl chitosan (CMCS) nanoparticles (ICED-N) targeted against the epidermal growth factor receptor (EGFR). (**A**) Radiochemical purity of ICED-N. (**B**) I-131 radioactive encapsulation efficiency of ICED-N. (**C**) Radioactive stability of ICED-N in normal saline. (**D**) Radiolabeling yield of ICED-N. Data presented are mean ± standard deviation (*n* = 3).

The Na^131^I radiolabeled ICED-N radiochemical purity (RCP) illustrated in Figure 2A indicates that the radiochemical purity over 3 days was >95%. In addition, the radioactive encapsulation efficiency (Figure 2B) after 1 day was ~98% and ~95% at 3 days, still within the 95% requirement set by the World Health Organization Consultation Document standard [38]. After 1 day, the ICED-N radioactivity stability was ~98% radioactive stability and ~96% after 3 days (Figure 2C). The radiolabeling yield (Figure 2D) indicates a good radiolabeling yield of 99.92% at 1 h and 99.2% at 12 h.

### 2.3. Quantification of Carboxymethyl Chitosan Nanoparticles (CMCS) and Anti-Human EGFR Antibody Surface Coverage

The determination of the nanoparticle surface density of the anti-EGFR ligands was conducted by spectrofluorimetry in order to quantify the Alexa Fluor 647-labeled anti-human EGFR nanoparticle coverage [39]. To confirm the presence of 50 mg of carboxymethyl chitosan (CMCS), a chitosan derivative with amphiprotic ether properties containing −COOH and −NH_2_ functional groups and solubility in water yielded around 5.54 × 10^19^ molecules × mg CMCS nanoparticle. This corresponds to a surface area of approximately 9.80 × 10^6^ nm^2^ and a volume of 4.09 × 10^8^ nm^3^ of 50 mg CMCS nanoparticles. Furthermore, applying 1 μg of anti-human EGFR antibody led to 4.49 × 10^12^ molecules of Alexa Fluor 647-labeled anti-human EGFR introduced onto the surface of nanoparticles. In addition, the bonding intensity between anti-EGFR ligands and nanoparticles was directly proportional to the number of ligands supplied during the production process (Table 1). This study utilized 50 mg of CMCS and 1 μg of anti-human EGFR antibody in the fabrication of the ICED-N. The quantification of carboxymethyl chitosan and anti-human EGFR antibody surface coverage calculations are shown in the supplement.

### 2.4. Entrapment and Loading Efficiency of Curcumin and Doxorubicin

Curcumin’s low bioavailability and water solubility hinder its therapeutic use; however, encapsulating in CMCS nanoparticles improves its solubility and stability. Curcumin entrapment efficiency was 85.48%, with a loading efficiency of 67.35%, and doxorubicin entrapment efficiency was 37.61%, with a loading efficiency of 42.26% (Table 2).

### 2.5. Release Profile and Mathematical Modelling of Release Kinetics of Curcumin- and Doxorubicin-Loaded ICED-N

#### 2.5.1. Release Profile of Curcumin- and Doxorubicin-Loaded ICED-N

**Figure 3:**
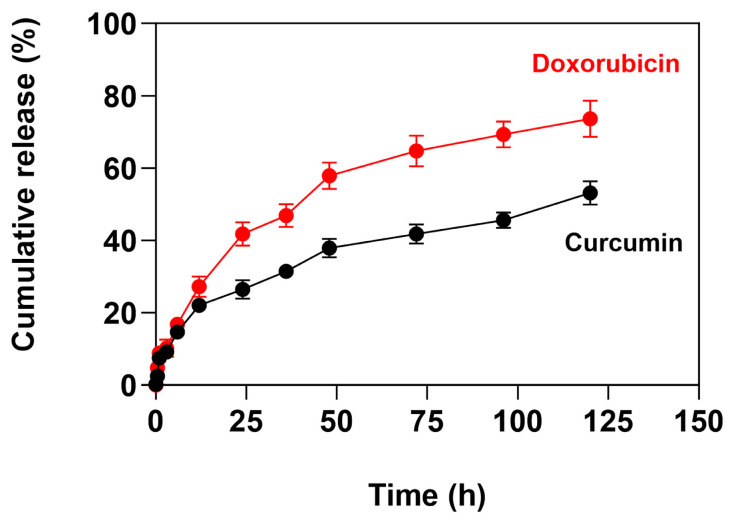
The co-administration release profile of doxorubicin and curcumin loaded within Na^131^I radiolabeled carboxymethyl chitosan (CMCS) nanoparticles (ICED-N) targeted against the epidermal growth factor receptor (EGFR) over a period of 120 h. Data presented are mean ± standard deviation (*n* = 3).

The ICED-N cumulative release of curcumin and doxorubicin was assessed at pH 7.4 over a period of 120 h (Figure 3). At 48 h, it was observed that ~58% of the doxorubicin was released, and at 120 h, ~74% was released. The drug release profile of nanoparticles can be illustrated through three primary phases: desorption, swelling, and erosion. The results confirm that the majority of the doxorubicin release occurs in 120 h, and the slower release after 48 h is because of the slower drug molecule diffusion through the chitosan matrix. The ICED-N CMCS molecular weight (MW) of 543.5 g/mol results in a deceased doxorubicin release than lower MW nanoparticles, as verified by Shi et al., who recorded an initial doxorubicin burst and a minor increase in drug release over 3 days [40,41]. Similarly, the in vitro release characteristics of ICED-N loaded with DOX exhibit an initial burst in the first 12 h, followed by a controlled release of DOX. The burst discharge has been attributed to DOX release from the NP outer surface and diffusion out of the ICED-N matrices that involve doxorubicin desorption and the polymeric CMCS nanoparticles. 

In addition, linear regression analysis of the doxorubicin release profile plotted against the square root of time resulted in a high coefficient of determination (R^2^ = 0.98). The curcumin release profile was similar to that of doxorubicin, although the percentage release was lower. At 24 h, ~26% was released, ~48% at 48 h, and ~53% at 120 h. The release data of curcumin and doxorubicin from ICED-N were analyzed using zero-order, Higuchi, and Korsmeyer–Peppas models to assess the processes and kinetics of release [42,43,44].

#### 2.5.2. Higuchi Mathematical Modelling of Release Kinetics of Curcumin- and Doxorubicin-Loaded ICED-N

**Figure 4:**
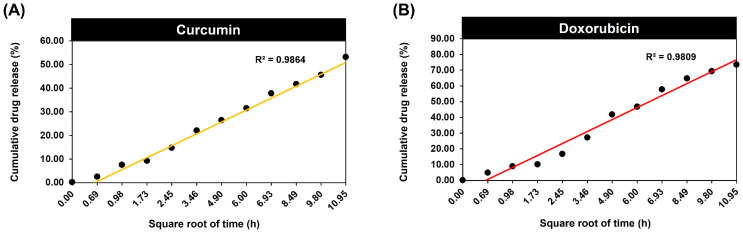
Higuchi mathematical modelling of release kinetics of (**A**) curcumin and (**B**) doxorubicin loaded within Na^131^I radiolabeled carboxymethyl chitosan (CMCS) nanoparticles (ICED-N) targeted against the epidermal growth factor receptor (EGFR) over a period of 120 h.

The Higuchi model is one of the most applied mathematical models for drug release kinetics. Drug release occurs from a permeable matrix when the amount of drug loaded exceeds its solubility limit, and enables accurate prediction of release rates [45]. The results of the Higuchi mathematical model determined a curcumin R^2^ value of 0.9864 and a doxorubicin R^2^ value of 0.9809 (Figure 4), indicating that Fickian diffusion and polymer relaxation mechanisms underlie the drug release process from the ICED-N. The accurate fitting of both curcumin and doxorubicin to the Higuchi kinetic release model suggests that the release is maintained during diffusion, as evidenced by the highest correlation coefficient value [46].

Out of the three drug release kinetic models applied (Figure 4, Figures S2 and S3, Tables S1 and S2), the Higuchi model was deemed better suited due to its minimal deviation from the experimental curve and the resulting release profile. The Higuchi model yielded a coefficient value, R^2^, which was very close to 1, suggesting that it is most suitable for accurately representing the release process of curcumin (R^2^ = 0.9864) and doxorubicin (R^2^ = 0.9809) from ICED-N.

### 2.6. Dose Response Cytotoxicity of Curcumin and Doxorubicin in Human Osteosarcoma Cell Proliferation

**Figure 5:**
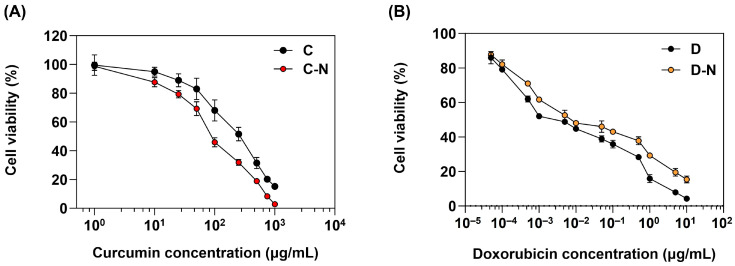
Dose response cytotoxicity of curcumin and doxorubicin on cell proliferation. (**A**) In vitro cytotoxicity of bare curcumin (C) and curcumin-loaded carboxymethyl chitosan nanoparticle (C-N) after 24 h incubation. (**B**) In vitro cytotoxicity of free doxorubicin (D) and doxorubicin-loaded carboxymethyl chitosan nanoparticle (D-N) after 24 h incubation. Data presented are mean ± standard deviation (*n* = 3).

A dose-response MTT assay in vitro cytotoxicity study investigated the impact of C and C-N on MG-63 osteosarcoma cells (Figure 5A). Additionally, Figure 5B illustrates the relationship between D and D-N concentrations and cell viability. The results confirmed that MG-63 cell development was inhibited by both C and C-N. A curcumin concentration of 100 μg/mL was loaded into the C-N, resulting in a viability of ~46%; in comparison, the same concentration of C resulted in ~68% viability, indicating that the C-N reduced the cell viability ~1.5-fold more than C. The greater toxicity of C-N relative to C could be due to the enhanced bioavailability achieved through nano-encapsulation [47]. Furthermore, the delayed release of C-N leads to a prolonged release, resulting in elevated curcumin concentrations and increased toxicity.

In addition, increasing the concentrations of C and C-N regulated the cell viability. The 50% inhibition concentration (IC_50_) value for C after 24 h treatment was 410 μg/mL and 156 μg/mL for C-N, verifying that the IC_50_ of C and C-N values were significantly different (*p* < 0.05). Additionally, the MTT assay in vitro study (Figure 5B) illustrates that the increase in doxorubicin concentration governs the rate of MG-63 cell inhibition. Furthermore, after 24 h of treatment, the IC_50_ D-N concentration of 6.58 μg/mL was similar to the D concentration of 6.49 μg/mL. Cell death or cell growth arrest is dependent on the doxorubicin concentration and length of treatment [48,49]. Rimann et al. investigated the biological significance of three-dimensional (3D) cell culture in the context of in vitro drug development [50]. Verifying that the 3D culture doxorubicin IC values were greater than the 2D culture values, the IC value for 3D Saos-2 was 0.3 μM, and 0.12 μM in the 2D culture.

### 2.7. Curcumin, Doxorubicin, and Radiation-Induced Cytotoxicity in 3D Human Osteosarcoma Spheroid 

**Figure 6:**
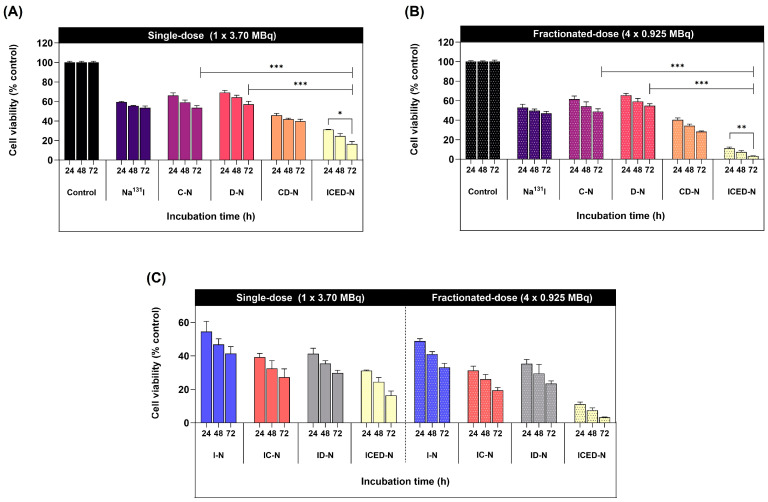
The co-administration cytotoxicity of doxorubicin and curcumin within Na^131^I radiolabeled carboxymethyl chitosan (CMCS) nanoparticles (ICED-N) epidermal growth factor receptor (EGFR) targeted against 3D human osteosarcoma spheroids. (**A**) Cell viability (% of control) treated with a single dose of control, Na^131^I, curcumin-loaded carboxymethyl chitosan nanoparticles (C-N), doxorubicin-loaded carboxymethyl chitosan nanoparticles (D-N), curcumin and doxorubicin-loaded carboxymethyl chitosan nanoparticles (CD-N), and co-administration of doxorubicin and curcumin within Na^131^I radiolabeled carboxymethyl chitosan nanoparticles (ICED-N) targeted against the epidermal growth factor receptor (EGFR) after 24, 48, and 72 h incubation. (**B**) Cell viability (% of control) treated with a fractionated dose of control, Na^131^I, C-N, D-N, CD-N, and ICED-N after 24, 48, and 72 h incubation. (**C**) Cell viability (% of control) treated with a single dose and a fractionated dose of I-N, IC-N, ID-N, and ICED-N after 24, 48, and 72 h incubation. Data presented are mean ± standard deviation (*n* = 3). * *p* ≤ 0.05, ** *p* ≤ 0.01, *** *p* ≤ 0.001.

The 3D MG-63 tumor spheroid liquid-based scaffold-free method, non-adherent surface stimulates cell-to-cell adhesion to form a 3D spheroid [51,52]. At 72 h, in the single-dose treatment group (Figure 6A), the C-N resulted in ~54% viability, and the D-N in ~57% viability. The CD-N combination achieved ~40% cell viability, and the ICED-N achieved ~16%. In comparison, the 4 × fractionated-dose treatment groups at 72 h achieved the following (Figure 6B). The C-N resulted in ~49% viability, and the D-N had a viability of ~55%. The CD-N resulted in ~28% viability, a 1.9-fold decrease in cell viability compared to the fractionated dose of D-N. Markedly, the fractionated-dose ICED-N resulted in significantly greater cytotoxicity, achieving ~3% cell viability (*p* ≤ 0.01). These results noticeably show that the ICED-N fractionated-dose regime resulted in cytotoxicity that is more significant to MG-63 cells than the single-dose treatment. Notably, at 72 h, the fractionated-dose ICED-N (4 × 0.925 MBq) achieved a ~5.3-fold reduction in MG-63 cell viability compared to the single-dose ICED-N. Having established that the fractionated-dose ICED-N treatment was the most effective, we assessed the efficacy of curcumin.

Figure 6C illustrates the viability of MG-63 cells after 72 h of single treatment with I-N, resulting in ~41% cell viability, and 72 h of 4 × fractionated-dose I-N treatments achieving ~33% cell viability. Markedly, the addition of curcumin to the platforms increased cytotoxicity, resulting in decreased cell viability. For instance, after 72 h of treatment with a single dose of IC-N, the cell viability was reduced to ~27%, a 1.2-fold decrease when compared to the I-N ~41% cell viability. Moreover, after 72 h of treatment with a single dose of ICED-N, the single dose achieved ~16% cell viability (*p* ≤ 0.05). In comparison, the fractionated ICED-N dose (4 × 0.925 MBq) after 72 h of treatment achieved a 5.3-fold more significant reduction of ~3% cell viability than the single-dose ICED-N (*p* ≤ 0.001). These results confirm that curcumin enhances Na^131^I cytotoxicity in MG-63 cells [53]. Additionally, curcumin augmented doxorubicin treatment is shown in Figure 6B. A fractionated dose of D-N resulted in ~55% viability, whereas the combination of CD-N resulted in a 1.9-fold more significant decrease in cell viability of ~28% (*p* ≤ 0.001). In fact, curcumin’s ability to improve doxorubicin absorption and decrease drug efflux in vivo has been validated by research led by Ma et al. [54].

### 2.8. Anti-Human EGFR Specifically Targets Human Osteosarcoma

**Figure 7:**
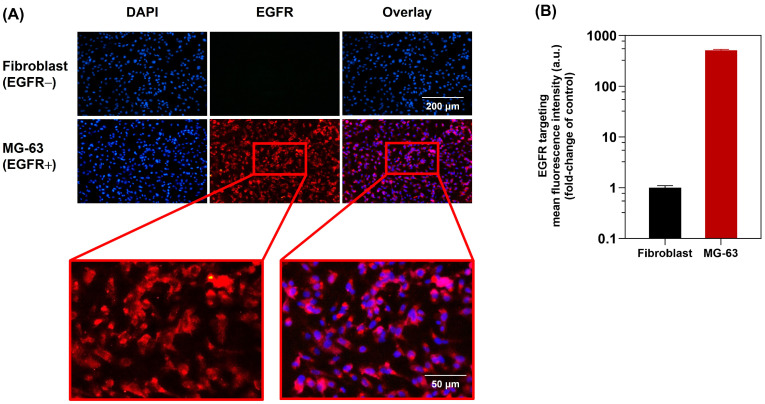
Co-localization of the co-administration of doxorubicin and curcumin within Na^131^I radiolabeled carboxymethyl chitosan (CMCS) nanoparticles (ICED-N) targeted against the epidermal growth factor receptor (EGFR) upon cellular binding/uptake. (**A**) In vitro anti-EGFR-targeted ICED-N immunofluorescence upon cellular binding/uptake of fibroblast (EGFR−) and MG-63 (EGFR+). Both the fibroblast (EGFR−) and MG-63 (EGFR+) cells were labeled with DAPI (blue channel), a fluorescent dye that has an affinity for DNA. Anti-EGFR labeled with Alexa Fluor 647 (red channel). (10× images, scale bar = 200 μm and 50 μm). (**B**) EFGR targeting mean fluorescence intensity (fold-change of control) of fibroblast (EGFR−) and MG-63 (EGFR+) cells. Data presented are mean ± standard deviation (*n* = 3).

The epidermal growth factor receptor (EGFR) is overexpressed in osteosarcoma and is a common genetic abnormality related to osteosarcoma [55]. Therefore, EGFR is a prospective therapeutic target in this research for the treatment of osteosarcoma. In order to evaluate the targeting of MG-63 cells, the anti-EGFR-targeted ICED-N immunofluorescence was measured by fluorescent live cell imaging microscopy to determine cellular binding/uptake to the MG-63 cells. Both the fibroblast (EGFR−) and MG-63 (EGFR+) cells were labeled with DAPI (blue channel), a fluorescent dye that has an affinity for DNA. Figure 7A illustrates fibroblast cells and the MG-63 nucleus stained blue. Additionally, conjugating Alexa Fluor 647 to anti-EGFR targeting ligands enabled the EGFR labelling efficacy to be visualized. As can be seen in Figure 7A, the ICED-N effectively targeted the MG-63 (EGFR+) cells (red channel), confirming that fluorescent live cell imaging verified good, effective binding to the MG-63 cells. In addition, there was minimal binding of the ICED-N to the control fibroblast (EGFR−) cells; in contrast, the MG-63 (EGFR+) cells revealed a strong fluorescence (red channel). As a result, the live cell imaging microscopy confirmed that the EGFR targeted ICED-N has a 513-fold greater targeting efficacy to MG-63 (EGFR+) cells than the control fibroblast (EGFR−) cells (Figure 7B).

### 2.9. Time-Dependent Study of Nanoparticle Penetration in 3D Human Osteosarcoma Tumor Spheroids

**Figure 8:**
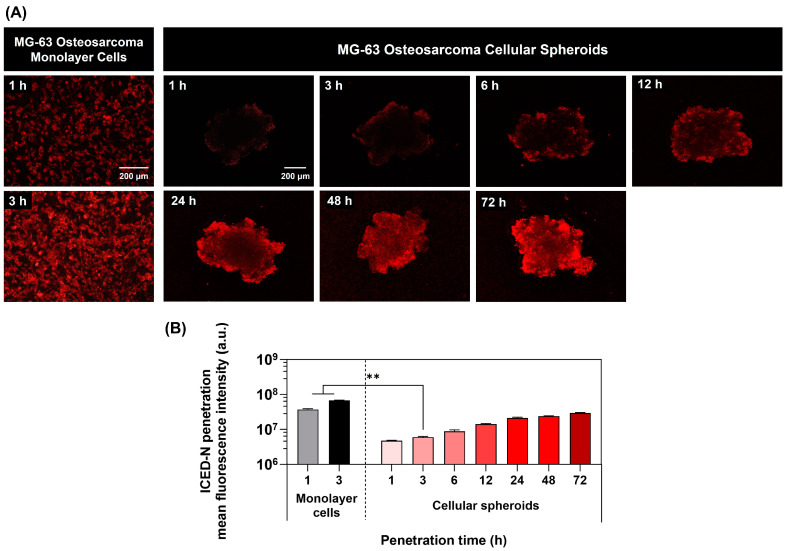
Time-dependent study of nanoparticle penetration in MG-63 human osteosarcoma. (**A**) The penetration of the co-administration of doxorubicin and curcumin within Na^131^I radiolabeled carboxymethyl chitosan (CMCS) nanoparticles (ICED-N) targeted against the epidermal growth factor receptor (EGFR) upon MG-63 osteosarcoma monolayer cells after 1 and 3 h incubation. The ICED-N penetration upon MG-63 osteosarcoma cellular spheroids after 1, 3, 6, 12, 24, 48, and 72 h incubation. Scale bar = 200 μm. Anti-EGFR labeled with Alexa Fluor 647 (red channel). (**B**) ICED-N penetration mean fluorescence intensity of MG-63 monolayer cells and MG-63 cellular spheroids. Data presented are mean ± standard deviation (*n* = 3). ** *p* ≤ 0.01.

The 3D MG-63 spheroid assessed the radiosensitization capacity of ICED-N, delivering a co-administration of curcumin and doxorubicin in the ICED-N. Previously, a comparison was conducted between the penetration of doxorubicin into monolayer cells (2D) and 3D MG-63 cellular spheroids, revealing that the doxorubicin penetration into the 3D spheroids occurred at a much slower rate in comparison to monolayer cells. At 1 h of treatment, the monolayer cells had an intracellular fluorescence intensity of 3.72 × 10^7^ a.u. In comparison, at 1 h, the fluorescent intensity of the 3D cells was 4.74 × 10^6^ a.u, revealing that the monolayer fluorescent intensity at 1 h treatment was 7.8-fold greater than the 3D cells. Furthermore, after 3 h treatment, the monolayer fluorescent intensity was ~11.3-fold greater than the 3D cells, confirming that the penetration of the less dense monolayer cells was quicker than the denser osteosarcoma spheroids. This study revealed that the utilization of a 3D MG-63 model with an artificial microenvironment is valuable for assessing the multicellular resistance that closely resembles the chemotherapy resistance often seen in solid tumors such as osteosarcoma [56]. At 72 h, the greatest degree of ICED-N penetration of the spheroids was detected (Figure 8A), illustrating that the intracellular fluorescence intensity of the ICED-N exhibits a pronounced fluorescence signal, suggesting significant penetration into the spheroid structure (*p* ≤ 0.01). The ICED-N mean fluorescence intensity after 72 h of treatment was ~3.4-fold greater than at 6 h treatment (Figure 8B).

### 2.10. Curcumin Enhances Radiation-Induced Cell Apoptosis/Programmed Cell Death Incidence Level in Human Osteosarcoma

**Figure 9:**
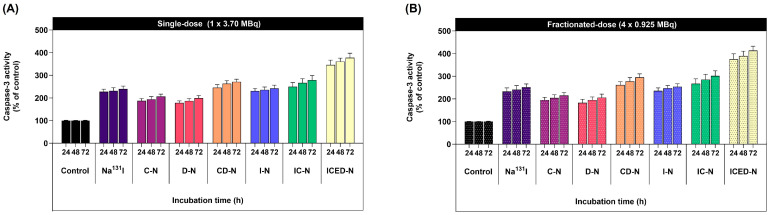
Cell apoptosis/programmed cell death incidence level of the co-administration of doxorubicin and curcumin within Na^131^I radiolabeled carboxymethyl chitosan (CMCS) nanoparticles (ICED-N) targeted against the epidermal growth factor receptor (EGFR). (**A**) Caspase-3 activity (% of control) treated with single-dose of control, Na^131^I, curcumin-loaded carboxymethyl chitosan nanoparticles (C-N), doxorubicin-loaded carboxymethyl chitosan nanoparticles (D-N), curcumin- and doxorubicin-loaded carboxymethyl chitosan nanoparticles (CD-N), Na^131^I radiolabeled carboxymethyl chitosan nanoparticles (I-N), Na^131^I radiolabeled and curcumin-loaded carboxymethyl chitosan nanoparticles (IC-N), and the co-administration of doxorubicin and curcumin within Na^131^I radiolabeled carboxymethyl chitosan nanoparticles (ICED-N) targeted against the epidermal growth factor receptor (EGFR) after 24, 48, and 72 h incubation. (**B**) Caspase-3 activity (% of control) treated with a fractionated dose of control, Na^131^I, C-N, D-N, CD-N, I-N, IC-N, and ICED-N after 24, 48, and 72 h incubation. Data presented are mean ± standard deviation (*n* = 3).

The caspase-3 activity fluorometric assay results are shown in Figure 9. The ICED-N fractionated-dose (4 × 0.925 MBq) caspase-3 activity was 1.1-fold greater than the ICED-N single-dose (3.70 MBq) caspase-3 activity after 72 h treatment. The caspase-3 activity of the single-dose ICED-N in comparison to the Na^131^I was 1.6-fold higher, and the caspase-3 activity of the Na^131^I was similar to the I-N (Figure 9A). However, the caspase-3 activity of the single-dose IC-N resulted in 1.2-fold higher programmed cell death than the I-N, suggesting curcumin increased radiosensitivity, inducing caspase-3 activation. In comparison, the 4 × fractionated-dose ICED-N (per fraction equivalent activity of 0.925 MBq) resulted in greater programmed cell death than Na^131^I single 3.70 MBq dose (Figure 9B). At 72 h, the 4 × fractionated-dose Na^131^I resulted in 1.05-fold greater programmed cell death than the single dose. Furthermore, the evaluation of curcumin enhanced treatment found that the fractionated-dose IC-N caspase-3 activity was 1.25-fold greater than the single-dose I-N, and 1.19-fold greater than the I-N (4 × fractionated-dose).

Moreover, the co-administration of curcumin with doxorubicin ICED-N achieved a pronounced increase in programmed cell death. For instance, the single-dose CD-N programmed cell death was 1.36-fold greater than the single-dose D-N. In addition, the 4 × fractionated-dose CD-N programmed cell death was 1.48-fold greater than the single-dose D-N. In summary, the findings from the fluorometric assay confirmed that curcumin enhanced doxorubicin treatment and Na^131^I radiosensitivity to MG-63 osteosarcoma cells.

### 2.11. Curcumin Enhances Doxorubicin- and Radiation-Induced G2/M Cell Cycle Arrest in Human Osteosarcoma

**Figure 10:**
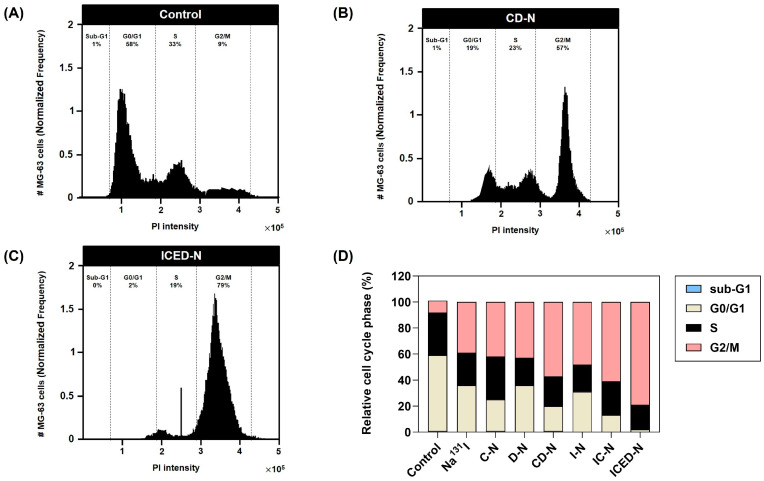
Relative cell cycle analysis in human osteosarcoma. The percentages of MG-63 human osteosarcoma in sub-G1, G0/G1, S, and G2/M treated cells was evaluated by the time-dependent influence of the combination formulations on cell cycle progression. Relative cell cycle phase (%) of MG-63 treated control, Na^131^I, curcumin-loaded carboxymethyl chitosan nanoparticles (C-N), doxorubicin-loaded carboxymethyl chitosan nanoparticles (D-N), curcumin- and doxorubicin-loaded carboxymethyl chitosan nanoparticles (CD-N), Na^131^I radiolabeled carboxymethyl chitosan nanoparticles (I-N), Na^131^I radiolabeled and curcumin-loaded carboxymethyl chitosan nanoparticles (IC-N), and the co-administration of doxorubicin and curcumin within Na^131^I radiolabeled carboxymethyl chitosan nanoparticles (ICED-N) targeted against the epidermal growth factor receptor (EGFR). (**A**) Control. (**B**) CD-N. (**C**) ICED-N. (**D**) Relative cell cycle phase (%) of control, Na^131^I, C-N, D-N, CD-N, I-N, IC-N, and ICED-N in sub-G1, G0/G1, S, and G2/M.

To determine the influence of curcumin on MG-63 cell cycle progression and apoptosis, the cells were treated for 72 h by control, Na^131^I (4 × fractionated-dose), fractionate dose C-N, fractionated-dose D-N, fractionated-dose CD-N, fractionated-dose I-N, fractionated-dose IC-N, and ICED-N. Treatment flow cytometer analysis specified the cell distribution percentages of the MG-63 cells (Figure 10). The untreated control MG-63 cell cycle profile has 1% sub-G1, 58% of cells in the G0/G1 phase, 33% in the S phase, and 9% in G2/M. After the treatments, there was a greater number of MG-63 cells in the G2/M phase, a decrease in G1 and the S phase. The transferal of the cell population from G1 to the G2 phase indicates enhanced radiosensitivity. The D-N treatment resulted in 43% cells in the G2/M phase and CD-N 57% in G2/M, a 1.33-fold increase. Furthermore, 48% of cells were in G2/M after I-N treatment. The IC-N resulted in 61% of cells in the G2/M phase, a 1.27-fold increase compared to the I-N (without curcumin).

In summary, the flow cytometer analysis confirms that the addition of curcumin to the radiolabeled nanoparticles increased radiosensitivity. This confirms that curcumin enhanced G2/M arrest and apoptosis when combined with the D-N and I-N to form CD-N and IC-N. Moreover, the ICED-N treatment resulted in 2% of cells in G0/G1, 19% in S phase, and 79% in G2/M. The cell distribution percentages verify that the ICED-N induced MG-63 cell arrest and apoptosis in the G2/M phase.

## 3. Discussion

High-grade osteosarcoma is an aggressive neoplasm that results in metastasis as first-line chemotherapy treatments suffer from drug resistance and, consequently, a poor prognosis [57,58]. Increasingly, acquired drug resistance has become more commonplace as a result of the tumor microenvironment (TME) changing after treatment, a second proto-oncogene becoming the new driver gene, or new mutations [59]. Acquired or intrinsic drug resistance in cancer cells can extensively impede the therapeutic efficacy of drugs and subsequently result in ∼90% therapeutic failure [60]. For this reason, substantial research has gone into surmounting resistance to develop strategies that overcome low therapeutic efficacy, low bioavailability, non-specific targeting, and toxicity [61].

In particular, research has demonstrated that plant phytochemicals like curcumin can modulate signaling pathways to limit cancer cell proliferation, migration, and metastasis, by preventing apoptosis and tumor angiogenesis, and reversing multidrug resistance [62,63]. Due to low bioavailability, poor dispersion, and quick metabolism in vivo, curcumin’s therapeutic uses are limited. Nevertheless, improvements in drug delivery, such as capsulated curcumin, specialized formulations, and nanoparticles, have made curcumin more bioavailable [64,65]. Research by Prasad et al. verified that curcumin-loaded chitosan nanoparticles are therapeutically effective against Vero cells [66]. Additionally, mucoadhesive chitosan nanoparticles were synthesized by Chuah et al. in order to transport curcumin to the colon and maintain its release, thereby enhancing its anticancer properties against colorectal cancer [67]. Equally, in our research, the radiolabeled fractionated-dose co-administration of curcumin- and doxorubicin-targeted carboxymethyl chitosan nanoparticle (ICED-N) reduced MG-63 cell viability 19-fold compared to the doxorubicin-loaded carboxymethyl chitosan nanoparticle (D-N). Moreover, co-administration therapeutics can improve outcomes, overcome resistance, and sustain therapy efficacy by targeting cancer cells from multiple angles [68].

Additionally, small curcumin molecules regulate cancer cell signaling pathways and suppresses drug-resistant protein synthesis [69], improving anti-tumor drug efficacy [48]. Furthermore, chemotherapy-resistant cells become more sensitive after being treated with curcumin, which reverses their resistance mechanisms [70]. Research by Wen et al. evaluated the effect of curcumin on doxorubicin chemoresistance in breast cancer cells, verifying curcumin reduced ABCB4-overexpressing cells efflux of doxorubicin, suppressing ABCB4 ATPase activity, which resulted in an increased accumulation of doxorubicin [71]. Notably, curcumin’s anticancer effects have been extensively investigated, revealing its ability to inhibit cancer cell proliferation, migration, and metastasis, induce apoptosis, hinder tumor angiogenesis, and reverse multidrug resistance through the regulation of signaling pathways. Targeting pathways with curcumin, such as MAPK/ERK, PI3k/AKT, Wnt/β-catenin, Notch, and MircoRNA, has been evaluated to improve osteosarcoma patient outcomes. In osteosarcoma, a key oncogenic factor is the PI3K/Akt pathway [11]. Additionally, the WNT pathway is another key factor in osteosarcoma development. However, its mechanism still remains unknown. Numerous studies have established a link between cancer and chronic inflammation [72]. Additionally, a review by Joshi et al. presents the complex chemical pathways and biological mechanisms by which curcumin and its equivalents exhibit their anti-cancer properties [73]. Chronic inflammation increases the formation of reactive oxygen species (ROS) and reactive nitrogen species (RNS), which causes DNA damage [74]. Cancer pathogenesis, chemoresistance, and radioresistance are all influenced by ROS dysregulation. Research by Xu et al. verified that curcumin increased the level of ROS in MG-63 cells, stimulating apoptosis [75]. In particular, WNT/β-catenin promotes cell invasion and migration in osteosarcoma, and WNT influences osteosarcoma chemotherapy resistance to cisplatin, doxorubicin, and methotrexate [76]. However, research by Wang et al. verified that curcumin impeded the Wnt/β-catenin pathway and non-small-cell lung cancer proliferation [77]. The upregulated WNT/β-catenin pathway in cancers exhibits a strong correlation with the presence of chronic inflammation and oxidative stress [78,79].

Nevertheless, osteosarcoma therapeutic approaches have seen little change, and in the past decade, there has been minimal improvement in the 5-year overall survival of patients, and the prognosis for patients who experience metastases or recurrence following treatment continues to be unfavorable [80]. The current standard of treatment for operable osteosarcoma patients is neoadjuvant chemotherapy, which typically involves the administration of doxorubicin, methotrexate, and cisplatin (MAP), followed by surgical intervention [81]. Osteosarcoma cells counteract chemotherapy cytotoxicity by reducing drug accumulation. Decreasing cell membrane folate carriers, increasing drug efflux, results in insufficient drug transport [82]. In addition, dysfunction lowering quality of life is common in osteosarcoma patients due to bone loss. Therefore, treatments having anti-cancer and pro-osteogenic characteristics are in great demand. A study by He et al. verified that curcumin could reverse the harmful effects of diabetic osteoporosis [83]. Additionally, a review by Ashrafizadeh et al. draws attention to the anticancer mechanism, side effects, and delivery of curcumin and doxorubicin by nanovesicles [84].

One notable characteristic of curcumin is its ability to regulate the initiation, development, and advancement of cancer, while simultaneously enhancing radiation and chemotherapy sensitivity. Furthermore, radioresistance is a term used to describe the capacity of cancer cells to endure the detrimental impacts of ionizing radiation, with numerous mechanisms and elements that influence radioresistance [85]. Glycolysis is an anaerobic reaction in the hypoxic TME rapidly consuming glucose and converting it to lactate, and is a key feature of cancer cells. A report by Kang et al. observed that radioresistant cancer cells are highly glycolytic and activate aerobic glycolysis in response to radiation damage. Since the Warburg effect is a characteristic of osteosarcoma and a metabolic feature that induces radioresistance, aerobic glycolysis is a possible therapeutic resistance target [86,87]. Of note, Siddiqui et al. found that glucose uptake and lactate production in cancer cells was inhibited by curcumin [88]. Additionally, curcumin may also improve radiosensitization, making radiation therapy more effective since it increases radiation-induced cell death (apoptosis) and inhibits cancer cell repair systems, rendering cancer cells more sensitive to radiation damage. This effect is thought to be due to curcumin’s ability to modulate various cellular processes, including inflammation, oxidative stress, and DNA repair mechanisms [81]. Curcumin enhances DNA damage to cancer cells, preventing them from growing and dividing, leading to significantly more damage to cancer cell DNA [89].

Additionally, drug carriers vary in form, size, drug dissolution profile, and functionality [90], making it impossible to create a universal release profile prediction model. Choosing the right model that matches each study situation is critical [91]. This research demonstrated that curcumin and doxorubicin were released from ICED-N via a diffusion-controlled mechanism. Due to its low divergence from the experimental curve and release profile, the Higuchi model was the best of the three kinetic models. Higuchi’s coefficient value, R^2^, was near to 1, suggesting it is best for representing the ICED-N’s curcumin (R^2^ = 0.9864) and doxorubicin (R^2^ = 0.9809) release. Similarly, Walbi et al. curcumin-loaded lecithin–Chitosan nanoparticles had a Higuchi’s coefficient value of 0.9876 [92]. Additionally, doxorubicin release from chitosan nanoparticles loaded with Dox followed the Higuchi model with an R^2^ of 0.9846 [93].

Furthermore, cancer cells form physical barriers that hinder host-immune responses and anti-cancer drugs from accessing the tumor. Cytokines and chemokines attract fibroblasts and myeloid cells to the TME, where they evolve into tumor-supporting cells, producing ECM proteins to protect malignant tumor cells. Despite dedifferentiation, malignant cells have epithelial connections that block the paracellular gap between tumor cells, safeguarding tumor antigens and target receptors. Most cancer treatment resistance is caused by the tumor’s ECM and epithelial junctions [94]. To closely mimic the TME, 3D human tumor spheroids are used as solid tumor models [95]. Cell-to-cell contact, proliferative gradients, hypoxia, and necrosis are possible in their 3D setup. The significance of human mesenchymal stem cells in ECM remodeling has been recognized due to their pro-tumorigenic function in the growth mechanisms of osteosarcoma tumors, which has led to their investigation of cell-mediated therapies [96]. Therefore, in this study, to better replicate the TME bone marrow, mesenchymal stem cells and human fibroblasts were cultured with the MG-63 cells to augment the 3D spheroid ECM [97]. Through multiphoton microscopy, we successfully generated three-dimensional representations of the characteristics of the MG-63 spheroids, allowing us to analyze ICED-N penetration, distribution, and uptake. The fluorescence signal inside the spheroid structure is prominently shown in Figure 8A, illustrating the penetration of ICED-N. Furthermore, this research verified that the 3D in vitro cytotoxicity 4 × fractionated-dose ICED-N (per fraction equivalent activity of 0.925 MBq) resulted in greater cytotoxicity than a single 3.70 MBq dose. A previous study also verified that a fractioned dose was more efficacious than a single dose [98]. In addition, in the 3D spheroid model, the EGFR-targeted ICED-N increased tumor accumulation with 513-fold greater targeting efficacy to MG-63 (EGFR+) cells than the control fibroblast (EGFR−) cells (Figure 7B). Pahl et al. studied the cytolytic activity of boosting natural killer to target sarcoma cells, and confirmed that EGFR was expressed by chemotherapy-resistant and chemotherapy-sensitive osteosarcoma cells [99].

Moreover, we verified that the Na^131^I fractionated dose caused 1.05-fold greater programmed cell death than the single dose. In addition, the fractionated-dose IC-N caspase activity was 1.25-fold higher than the single-dose I-N and the co-combination of curcumin and doxorubicin ICED-N significantly increased programmed cell death (Figure 9B). Additionally, in a study by Mbese et al., curcumin impeded the cell cycle and reduced cyclin-dependent kinase expression, which can result in cancer cell proliferation [82]. According to a study conducted by Wang et al., curcumin induces G2/M cell cycle arrest and cellular senescence in cervical cancer cell lines and was dependent on the dosage of curcumin administered, resulting in reduced cell survival [100]. Similarly, this study confirmed that the administration of curcumin resulted in enhanced effectiveness of doxorubicin and greater radiosensitivity to Na^131^I. The irradiation of MG-63 cells with curcumin-enhanced radiolabeled nanoparticles increased the proportion of cells in the G2/M phase. This observation suggests the damaged DNA was being repaired [101]. Additionally, it was confirmed that the fractionated irradiation dose caused more significant DNA damage to the cells than a single dose, as depicted in Figure 10.

## 4. Materials and Methods

### 4.1. Materials and Cell Lines

MG-63 human osteosarcoma cell line, human fibroblasts, and bone marrow mesenchymal stem cells were acquired from the American Type Culture Collection (ATCC), situated in Manassas, VA, USA. The MG-63 cells, human fibroblasts and bone marrow mesenchymal stem cells were grown at a temperature of 37 °C in a humidified atmosphere (5% CO_2_ and 95% air environment). The MG-63 and fibroblasts culture media employed in this study consisted of Dulbecco’s Modified Eagle’s media, supplemented with 10% fetal bovine serum and 1% penicillin/streptomycin. The bone marrow mesenchymal stem cells were supplemented with 16.5% fetal bovine serum and 1% penicillin/streptomycin. The cell lines were subjected to a minimum of four subcultures using trypsin/ethylenediaminetetraacetic acid (trypsin/EDTA) treatment and maintained in a humidified atmosphere (5% CO_2_ and 95% air environment). The medium was refreshed periodically every 3 days. The cells were introduced to the 3D low attachment plates at a concentration of 5 × 10^3^ − 1 × 10^4^ cells/mL.

### 4.2. Preparation and Characterization of Nanoparticles 

In brief, the process for synthesizing the various treatments is described in Figure 1A, and outlined as follows:

- Control: The Dulbecco′s Modified Eagle (DMEM) complete medium was composed of DMEM high glucose medium supplemented with 2 mM L-glutamine, sodium pyruvate, 10% fetal bovine serum (FBS), and 1% penicillin/streptomycin.
- CMCS: The preparation of carboxymethyl chitosan was conducted using the method proposed by Sun et al. [102]. A mixture consisting of 10 g of chitosan, 10 g of sodium hydroxide, 50 mL of isopropanol, and 50 mL of water was introduced into a flask for the purpose of swelling and alkalization. The process was carried out at a temperature of 50 °C for a duration of 1 h. A solution of monochloroacetic acid (15 g) was prepared by dissolving it in isopropanol (20 mL). This solution was then added drop-wise to the reaction mixture over a period of 30 min and allowed to react for a duration of 4 h at 50 °C. The reaction was thereafter terminated by the addition of 70% ethyl alcohol (250 mL). The solid material underwent filtration and subsequent rinsing using ethyl alcohol with a concentration ranging from 70% to 90%. Following this, the solid was subjected to vacuum drying at room temperature. The product under consideration is the sodium salt of carboxymethyl chitosan (Na-CC). Na-CC suspension (1 g) was prepared in a 100 mL solution of 80% ethyl alcohol in water. Subsequently, 10 mL of 37% hydrochloric acid was added to the suspension, and the mixture underwent stirring for a duration of 30 min. The solid material underwent filtration and subsequent rinsing in ethyl alcohol with a concentration of 70−90% in order to achieve neutralization. Following this, the solid was subjected to vacuum drying [103].
- Na^131^I: Sodium iodide, dissolved in 1 M phosphate-buffered saline (1 × PBS), with an activity of 3.70 MBq (100 μCi).
- C-N: The double emulsion procedure utilized to prepare the CMCS cores, into which curcumin was inserted into the inner phase, curcumin was diffused using 25 μL of 1 × PBS. The diffusion process was followed by sonication for 2 min at 70% pulsed power, with a pattern of 2 s on, and 1 s off. The core material employed during sonication was CMCS. Subsequently, the solution was combined with 5 mL of 1 × PBS and subjected to sonication for 2 min. Next, a volume of 10 mL of 1 × PBS was introduced to facilitate the process of evaporation. The mixture was then agitated for 4 h within a fume hood.
- D-N: Doxorubicin was loaded into the CMCS core using the double emulsion, following the same procedure as the C-N.
- CD-N: Curcumin and doxorubicin were loaded into the CMCS cores using the double emulsion procedure, following the same procedure as the C-N.
- I-N: Na^131^I was loaded into the CMCS cores using the double emulsion procedure without chelating agents, following the same procedure as the C-N.
- IC-N: Na^131^I and curcumin were loaded into the CMCS cores using the double emulsion procedure, following the same procedure as the C-N.
- ID-N: Na^131^I and doxorubicin were loaded into the CMCS cores using the double emulsion procedure, following the same procedure as the C-N.
- ICD-N: Na^131^I, curcumin, and doxorubicin were loaded into the CMCS cores using the double emulsion procedure.
- ICED-N: The double emulsion method was used to synthesize the epidermal growth factor receptor (EGFR)-targeted Na^131^I radiolabeled carboxymethyl chitosan (CMCS) nanoparticle, with co-administration of doxorubicin and curcumin (ICED-N).

In addition, the physiochemical characteristics, size, polydispersity, and zeta potential of ICED-N were assessed by dynamic light scattering (DLS) evaluations conducted in triplicate (*n* = 3) at room temperature. The Malvern ZEN 3600 Zetasizer instrument was utilized for this investigation. Furthermore, Alexa Fluor 647 was employed to validate the interaction between the anti-human EGFR antibody and the co-administration of doxorubicin and curcumin within the Na^131^I radiolabeled carboxymethyl chitosan (CMCS) nanoparticle (ICED-N) targeted against the epidermal growth factor receptor (EGFR) and the uptake of these nanoparticles by MG-63 osteosarcoma (EGFR+). The fluorescence intensity was evaluated using a LionHeart FX Automated Microscope (BioTek Instrument, Winooski, VT, USA) for live cell imaging [104].

### 4.3. Radioactive Properties of Nanoparticles 

#### 4.3.1. Radiochemical Purity 

The standard radiochemical purity percentage (% RCP) of I-131 was determined using instant thin-layer chromatography (ITLC) provided by Global Medical Solutions, Bangkok, Thailand. The quantity of unbound I-131 was measured using a dosage calibrator manufactured by Capintec, Inc., Ramsey, NJ, USA. Furthermore, the presence of impurities did not exceed 5% of the overall activity [38]. The radiochemical purity was obtained using Equation (1):

(1) $$Radiochemical\;Purity\;(RCP)=\left[\frac{Total\;Activity-Impurities\;Activity}{Total\;Activity}\right]\times 100\%$$

#### 4.3.2. Radioactive Stability 

ICED-N were incubated at a temperature of 37 °C in 1 × PBS. The ITLC-SG method was employed to evaluate the stability of the ICED-N in nine consecutive serum samples using a 0.9% NaCl solution as the eluent. In order to determine their stability, the alteration in the ICED-N and the radioactive stability were assessed in triplicate for 72 h (*n* = 3).

#### 4.3.3. Radioactive Yield 

The yield of I-131 radiolabeling was assessed by ITLC-SG after the development of ICED-N in a 0.25% KCl chloroform–methanol solution at various time intervals ranging from 0 to 72 h. Retardation or Retention Factor ($R_{f}$) measurements refer to the distance traveled by a substance relative to the solvent and are used for the purpose of identifying the locations of I-131 radioactive areas [105]. Bound ICED-N exhibits a migration pattern at $R_{f}$ = 0.0 during the migration process. However, unbound I-131 migrates towards the solvent front region of the ITLC strip, which is between $R_{f}$ = 0.6 and 1.0 [106].

### 4.4. Entrapment and Loading Efficiency of Curcumin and Doxorubicin

#### 4.4.1. Entrapment and Loading Efficiency of Curcumin

In order to assess the entrapment effectiveness of curcumin inside the ICED-N, a process of ultracentrifugation was employed. The ultracentrifugation was carried out at a speed of 20,000 rpm and a temperature of 4 °C for 45 min. This separation process aimed to isolate the ICED-N from the aqueous medium, which included curcumin that was not encapsulated. Following two rounds of washing with distilled water, the pellet underwent re-dispersion in ethanol. The resulting mixture was thoroughly vortexed and subsequently subjected to centrifugation [107]. A yellowish supernatant was obtained from this process, which was then measured using a consistent UV absorption wavelength of 467 nm and fluorescence emission at 571 nm [108]. Determining loading efficiency was further performed by considering the mass of the nanoparticles acquired during the centrifugation process.

(2) $$Entrapment\;efficiency\;of\;curcumin=\frac{Amount\;of\;curcumin\;in\;the\;ICED-N\;pellet}{Initial\;amount\;of\;curcumin}\times 100\%$$

(3) $$Loading\;efficiency\;of\;curcumin=\frac{Total\;amount\;of\;curcumin}{Yield\;of\;ICED-N}\times 100\%$$

#### 4.4.2. Entrapment and Loading Efficiency of Doxorubicin

To evaluate the efficacy of doxorubicin entrapment within ICED-N, an ultracentrifugation technique was utilized. The ultracentrifugation procedure was conducted at a rotational speed of 20,000 rpm at 4 °C for 45 min. The objective of this separation procedure was to extract the ICED-N compound from the aqueous solution, which included unencapsulated doxorubicin. The pellets were subjected to two cycles of washing using distilled water, after which they were re-dispersed in ethanol. The resultant mixture was vigorously agitated using a vortex mixer and later underwent centrifugation. The resulting solution from this procedure exhibited a reddish supernatant, which was subsequently quantified using a consistent UV absorption wavelength of 480 nm and red fluorescence emission at 580 nm. The assessment of loading efficiency was further conducted by taking into account the mass of the nanoparticles obtained after the centrifugation procedure.

(4) $$Entrapment\;efficiency\;of\;doxorubicin=\frac{Amount\;of\;doxorubicin\;in\;the\;ICED-N\;pellet}{Initial\;amount\;of\;doxorubicin}\times 100\%$$

(5) $$Loading\;efficiency\;of\;doxorubicin=\frac{Total\;amount\;of\;doxorubicin}{Yield\;of\;ICED-N}\times 100\%$$

### 4.5. Release Profile of Curcumin- and Doxorubicin-Loaded ICED-N

The investigation into the release of curcumin- and doxorubicin-loaded ICED-N over time was carried out using Slide-A-Lyzer MINI Dialysis Cups with a 3.5 kDa molecular weight cut-off. These cups were placed in a sealed beaker containing 2000 mL of 1 × PBS with a pH of 7.4, which served as the release medium. The contents were agitated at 200 rpm and maintained at a temperature of 37 °C. At specific time intervals, samples were collected from the buffer solution, and an equivalent volume of fresh buffer medium was added to ensure a constant pH and sink state. To extract curcumin and doxorubicin from ICED-N, a solution of 0.1 M HCl in acetonitrile was used. The concentration of doxorubicin was determined by measuring its fluorescence, with excitation at 480 nm and emission at 580 nm. Likewise, the concentration of curcumin was determined by measuring its fluorescence, with excitation at 467 nm and emission at 571 nm. The experiments were carried out in triplicate, and the results are presented as the average of these three measurements (mean ± standard deviation) (*n* = 3).

(6) $$Drug\;release\;of\;curcumin=\frac{Curcumin\;released\;at\;a\;specific\;time\;interval}{Total\;amount\;of\;curcumin\;entrapped\;within\;ICED-N}\times 100\%$$

(7) $$Drug\;release\;of\;doxorubicin=\frac{Doxorubicin\;released\;at\;a\;specific\;time\;interval}{Total\;amount\;of\;doxorubicin\;entrapped\;within\;ICED-N}\times 100\%$$

### 4.6. Drug Release Mathematical Models

The release of curcumin and doxorubicin from ICED-N can be analyzed using various mathematical models. The release data fitting was performed using MATLAB 2007 software. After analyzing the regression coefficient, the optimal kinetic model was determined. This research focused on investigating the controlled release kinetics utilizing the zero-order [109], Higuchi model [46], and Korsmeyer–Peppas models [110]. The normalized curcumin and doxorubicin release from ICED-N at 0, 0.5, 1, 3, 6, 12, 24, 36, 48, 72, 96, and 120 h calculated using the following equations:

#### 4.6.1. Zero-Order Release: Assumes a Constant Release Rate over Time

(8) $$C_{t}=C_{0}+(K_{0}\times t)$$

where $C_{t}$ is the drug released amount at time = t, $C_{0}$ is the initial drug concentration at time t = 0, and $K_{0}$ is the zero-order rate constant (concentration/time).

#### 4.6.2. Higuchi Model: Describes Drug Release from a Matrix System Where Release Is Diffusion-Controlled

(9) $$Q=K_{H}\times t^{1/2}$$

where Q is the drug released cumulative amount in time = t per unit area, and $K_{H}$ is the Higuchi dissolution constant.

#### 4.6.3. Korsmeyer–Peppas Model (Power Law): Suitable for Showing Anomalous (Non-Fickian) Release Kinetics, a Combination of Diffusion and Polymer Relaxation

(10) $$log\;\frac{M_{t}}{M_{\infty }}=logK_{kp}+n\log t$$

where $M_{t}$ is the quantity of drug released in the time = t, $M_{\infty }$ is the amount of drug released after time = ∞, $K_{kp}$ is the Korsmeyer–Peppas release rate constant, and n is the diffusional exponent or the drug release exponent.

### 4.7. In Vitro Dose Response Cytotoxicity of Curcumin and Doxorubicin in Human Osteosarcoma Cell Proliferation: MTT Assay

#### 4.7.1. In Vitro Dose Response Cytotoxicity of Curcumin in Human Osteosarcoma Cell Proliferation

The MTT assay is a method employed to evaluate cells’ metabolic activity and vitality, utilizing a colorimetric approach to quantify cellular metabolic activity. The process relies on the enzymatic activity of nicotinamide adenine dinucleotide phosphate (NADPH)-dependent cellular oxidoreductase enzymes, catalyzing the reduction of the tetrazolium dye MTT. This results in an insoluble formazan compound with a distinct purple hue. The assay quantifies cell viability by assessing the enzymatic conversion of a tetrazolium compound to water-insoluble formazan crystals, facilitated by dehydrogenases primarily found in the mitochondria of viable cells. Notably, reducing agents and enzymes present in other organelles, such as the endoplasmic reticulum, also contribute to this process [111,112].

In assessing therapeutic effectiveness in a controlled laboratory setting, the MTT assay was employed. MG-63 osteosarcoma cells were cultured in Dulbecco’s Modified Eagle Medium (DMEM) (Gibco-BRL, Waltham, MA, USA), supplemented with 10% FBS, 1% penicillin/streptomycin (Gibco-BRL, Waltham, MA, USA), and L-glutamine (Gibco-BRL, Waltham, MA, USA). Initially, MG-63 cells were cultivated until they reached a confluence of 60–80% at 37 °C and a 5% CO_2_ in culture flasks within a humidified incubator. Subsequently, 5000 cells per well were seeded into 96-well microtiter plates, with a final volume of 200 µL per well, 24 h before initiating therapy.

Next, a group of MG-63 cells (*n* = 3) underwent treatment with free curcumin (C) or C-N at concentrations of 0, 1, 10, 25, 50, 100, 250, 500, 750, and 1000 µg/mL for 24 h. The C and C-N underwent a washing process and was subsequently cultured in a fresh culture mixture for another 24 h, with each well containing a final volume of 200 µL. The rate of cell proliferation was assessed using the MTT assay, involving the incubation of a mixture of 50 μL of serum-free media and 50 µL of MTT solution in 96-well plates for 3 h at 37 °C under a 5% CO_2_ atmosphere. Following the addition of 150 µL of MTT solvent to each well, the well plates were covered with aluminum foil to prevent light passage and stirred for 15 min using an orbital shaker. The absorbance of the treated cells was determined using a microplate reader set to an optical density (OD) of 590 nm, establishing the correlation between the color intensity observed and the quantity of live cells.

#### 4.7.2. In Vitro Dose Response Cytotoxicity of Doxorubicin in Human Osteosarcoma Cell Proliferation

MG-63 osteosarcoma cells were cultured in Dulbecco’s Modified Eagle Medium (DMEM) supplemented with 10% FBS, 1% penicillin/streptomycin, and L-glutamine at 37 °C and a 5% CO_2_ in culture flasks within a humidified incubator. At 24 h prior to the treatment, 5000 cells per well were inoculated into 96-well microtiter plates (200 µL per well) once cells had attained 60−80% confluence.

Following that, MG-63 cells (*n* = 3) were subjected to free doxorubicin (D) or D-N treatment for 24 h at 5 × 10^−5^, 1 × 10^−4^, 5 × 10^−4^, 1 × 10^−3^, 5 × 10^−3^, 1 × 10^−2^, 5 × 10^−2^, 1 × 10^−1^, 5 × 10^−1^, 1, 5, and 10 µg/mL concentrations. Following the rinsing of D or D-N, cells were cultured for an additional 24 h in a fresh mixture containing 200 μL per well. Cell proliferation was evaluated using the MTT assay, which entailed a 24 h incubation at 37 °C with 5% CO_2_ of a 50 μL mixture of serum-free media and MTT solution in 96-well microtiter plates. Plates were covered with aluminum foil and agitated for 15 min with an orbital shaker, and absorbance was measured at 590 nm after 150 µL of MTT solvent was added to each well. The correlation between observed color intensity and viable cell quantity was ascertained in this manner.

### 4.8. In Vitro Cytotoxicity in Human Osteosarcoma Spheroids 

MG-63 human osteosarcoma 3D spheroids were treated for 24, 48, and 72 h with single-dose or 4 × fractionated-dose of control, Na^131^I, C-N, D-N, CD-N, and ICED-N. A 3D Cell Titer-Glo^®^ cell viability assay was used to investigate the survival of the MG-63 cells. Following the manufacturer’s instructions, we discarded the medium and added 100 μL of Cell Titer-Glo^®^ reagent to each well, mixed for 2 min, then incubated for 30 min at room temperature. A plate reader (Tecan, Infinite 200 PRO, Männedorf, Switzerland) was used to analyze the luminescence signal.

### 4.9. In Vitro Surface Immunofluorescence Cellular Binding in Human Osteosarcoma

The control fibroblast cells (EGFR−) utilized in this study were characterized as aged cells, which have a lower EGFR expression, as opposed to young cells, which exhibit elevated levels of EGFR. Using younger fibroblast cells would distort the intracellular targeting evaluation [113]. Intercellular binding/uptake of MG-63 (EGFR+) and fibroblast (EGFR−) cells (*n* = 3) was determined by incubating them with ICED-N for 24 h. First, the MG-63 and fibroblast cells were rinsed with 1 × PBS, then treated with 4% paraformaldehyde for 20 min to preserve samples. Then, they were stained with 4′,6-diamidino-2-phenylindole (DAPI) dye (blue channel) for 30 min at 37 °C and rinsed twice before fluorescent live cell imaging microscopy examination.

### 4.10. Time-Dependent Study of Nanoparticle Penetration in MG-63 Osteosarcoma Monolayer Cells and MG-63 Osteosarcoma Cellular Spheroids

The evaluation of ICED-N penetration capability utilized MG-63 monolayer cells. Qualitative and quantitative assessment of ICED-N penetration into 2D monolayer MG-63 cells was performed using multi-photon fluorescence microscopy. ICED-N was administered at 0, 1, and 3 h. Throughout the exposure period, MG-63 cells underwent thorough washing and rinsing with 1 × PBS before fixation in a solution of 1 × PBS containing 4% paraformaldehyde for 60 min. Subsequently, cells were washed, stained with DAPI, and visualized using LionHeart live cell imaging. The quantification of ICED-N penetration was conducted by measuring fluorescence intensity.

Moreover, the MG-63 cellular 3D spheroid model ~600 µm diameter was utilized to assess the penetration capability of ICED-N. Multi-photon fluorescent microscopy then assessed the entry of ICED-N into 3D MG-63 cellular spheroids both qualitatively and quantitatively. The radiolabeled ICED-N treated one spheroid per well for 6, 12, 24, 48, and 72 h. Throughout 6, 12, 24, 48, and 72 h exposure to ICED-N, the spheroids were washed and rinsed with 1 × PBS before being fixed in 1 × PBS containing 4% paraformaldehyde for 60 min. Afterward, the spheroids were washed, then dyed with DAPI dye before being imaged using LionHeart live cell imaging and their fluorescence intensity quantified the progression of ICED-N penetration.

### 4.11. Apoptosis/Programmed Cell Death Incidence Level in Human Osteosarcoma

Initially, the MG-63 cells were placed at a density of 1 × 10^5^ to 1 × 10^6^ cells per well in a complete DMEM medium. Subsequently, the cells were grown in a controlled environment with 5% CO_2_ at 37 °C for 24 h. Next, the cells underwent a 72 h treatment period with single-dose and 4 × fractionated-dose of control, Na^131^I, C-N, D-N, CD-N, I-N, IC-N, and ICED-N. Following the completion of the treatment, the supplemented DMEM medium was taken out, and the cells were rinsed with 1 × PBS, then dissociated using trypsin/EDTA to eliminate contaminants and achieve a state of individual cell suspension. The caspase-3 activity induced by the therapies was evaluated using a fluorometric assay kit (ab39383, Abcam, Cambridge, UK). Following the cells’ collection, they were re-suspended in a cooled Lysis Buffer I/Cell Lysis Buffer (50 μL). The cells were then subjected to a period of 10 min on ice. Subsequently, a volume of 50 μL of Reaction Buffer I/2 (containing 10 mM DTT II/DTT) was added to each sample. The cells that underwent treatment were subjected to incubation with a 1 mM DEVD-AFC substrate, with a final concentration of 50 μM, in a volume of 5 μL. Subsequently, the cell fluorescence measured using a fluorescence microtiter plate reader equipped with a 400 nm excitation filter and a 505 nm emission filter [98].

### 4.12. Relative Cell Cycle Analysis in Human Osteosarcoma

The assessment of cell cycle analysis involved the following steps: trypsin dissociation of MG-63 cells, subsequent rinsing with an assay buffer to maintain solution equilibrium, and suspension of cell pellets in a new assay solution at a concentration of 1 × 10^6^ cells/mL. Subsequently, in preparation for propidium iodide labeling, cellular permeabilization was achieved by treating the cells with 1000 μL of a cell fixation agent, thus halting any ongoing metabolic activities. The fixing agent was eliminated using a centrifugation process lasting 5 min at a force of 500× *g*. Subsequently, the cells were immersed in a propidium iodide staining solution and afterwards subjected to incubation in a light-deprived environment at room temperature for a duration of 30 min. The MG-63 cell cycle was analyzed using the ImageStreamX Mk II flow cytometer (Merck KGaA, Darmstadt, Germany).

### 4.13. Statistical Analysis

In this investigation, each in vitro experiment was performed three times independently. The in vitro experiments yielded data expressed as a mean ± standard deviation. In addition, the residual was used to assess the normality of the data, and the variance similarity between groups was determined by analyzing the variance of each group. A Student’s *t*-test and one-way or two-way analysis of variance (ANOVA), followed by a student Newman–Keuls post hoc test, were employed to determine the statistical significance. The ns: *p* > 0.05, * *p* ≤ 0.05, ** *p* ≤ 0.01, *** *p* ≤ 0.001, **** *p ≤* 0.0001 were utilized to demonstrate statistical significance. GraphPad Prism 10.0 software (GraphPad Software Inc., Boston, MA, USA) was utilized for all statistical analyses. 

## 5. Conclusions

The primary objective of this study was to assess the efficacy of the small molecule curcumin in enhancing the effects of chemotherapy and radiation therapy against MG-63 osteosarcoma cells. The ICED-N delivers a co-administration treatment of curcumin and doxorubicin loaded in a carboxymethyl chitosan (CMCS) nanoparticle. Directly labeled with Na^131^I, it is the first nanoplatform of its type to treat osteosarcoma cells in a 3D spheroid model. Characterization revealed the ICED-N with a mean diameter of ~257 nm, a PDI of 0.22, and a zeta potential of −12 mV. With a curcumin entrapment efficiency of ~85% and a loading efficiency of 67%, and a doxorubicin entrapment efficiency of ~38% and a loading efficiency of ~42%. The hydrophilic Na^131^I radiolabeled without chelating agents, enabling the delivery of larger radioactivity doses. The radiolabeling resulted in ~98% radioactive stability and a radiolabeling yield of 99.92% at 1 h. The anti-EGFR-targeted ICED-N immunofluorescence confirmed a 513-fold greater targeting efficacy to MG-63 (EGFR+) cells than the control fibroblast (EGFR−) cells. The ICED-N, 4 × fractionated-dose regime resulted in a significant 18.3-fold greater cytotoxicity to the 3D MG-63 cells than the single-dose doxorubicin-loaded nanoparticle (D-N). Furthermore, the 3D MG-63 spheroid model proved to be a significant tool in evaluating multilayer resistance inside osteosarcoma tumors. Our results demonstrate that the ICED-N exhibited a substantial level of penetration into the spheroid structure. In addition, the combination of curcumin and doxorubicin resulted in a significant increase in programmed cell death. The ICED-N fractionated-dose caspase-3 activity was 2.1-fold greater than the single-dose doxorubicin-loaded nanoparticle (D-N). Moreover, the results of this investigation confirmed that the inclusion of curcumin enhanced doxorubicin effectiveness and increased radiosensitivity to Na^131^I. When exposed to Na^131^I radiation, the curcumin-enhanced radiolabeled nanoparticles increased the proportion of MG-63 cells in the G2/M phase 8.78-fold, and a 58-fold reduction in the G0/G1 phase.

Moreover, this radio-nanotherapeutic platform effectively administered a combination of curcumin and doxorubicin fractionated radiotherapeutic, successfully inhibiting MG-63 osteosarcoma cells. This radiotherapeutic platform holds promise as a potential platform for the advancement of osteosarcoma therapy and diagnosis in the future. The use of nanotheranostics has facilitated significant advancements; however, there are still some significant obstacles that are unique to nanotheranostics [114]. In the process of advancing bench to bedside radio-nanotherapeutics, it is imperative to investigate a multitude of issues, including adverse effects, compatibility, compliance, regulatory approvals, reliability, repeatability, scalability, and commercial possibilities, and the long-term effects of bone-seeking nanomedicines need to be resolved.

## Figures and Tables

**Table 1:** Quantification of carboxymethyl chitosan (CMCS) nanoparticles and anti-human EGFR antibody surface coverage. The determination of surface area (SA) of nanoparticle, volume of nanoparticle, SA:Volume ratio, molecules of nanoparticle, and number of CMCS particles was conducted by employing surface area and volume approximations for spherical nanoparticles. The data presented in this study represent the mean value and its corresponding standard deviation (mean ± SD) (*n* = 3).

Carboxymethyl Chitosan (CMCS) Nanoparticles					
Conjugation (mg)	Surface Area (SA) (nm^2^ × mg NP)	Volume (nm^3^ × mg NP)	SA:Volume Ratio (nm^2^/nm^3^)/mg NP	Molecules × mg NP	Number of CMCS Particles
1	1.96 × 10^5^	8.18 × 10^6^	2.40 × 10^−2^	1.11 × 10^18^	3.50 × 10^9^
5	9.80 × 10^5^	4.09 × 10^7^	4.79 × 10^−3^	5.54 × 10^18^	1.75 × 10^10^
10	1.96 × 10^6^	8.18 × 10^7^	2.40 × 10^−3^	1.10 × 10^19^	3.50 × 10^10^
25	4.90 × 10^6^	2.05 × 10^8^	9.58 × 10^−4^	2.77 × 10^19^	8.75 × 10^10^
50	9.80 × 10^6^	4.09 × 10^8^	4.79 × 10^−4^	5.54 × 10^19^	1.75 × 10^11^
100	1.96 × 10^7^	8.18 × 10^8^	2.40 × 10^−4^	1.11 × 10^20^	3.50 × 10^11^
200	3.92 × 10^7^	1.64 × 10^9^	1.20 × 10^−4^	2.22 × 10^20^	7.00 × 10^11^

**Table 2:** Entrapment and loading efficiency of curcumin and doxorubicin.

Drug	Entrapment Efficiency (%)	Loading Efficiency (%)
Curcumin	85.48	67.35
Doxorubicin	37.61	42.26

## Data Availability

Access to detailed data is restricted due to both ethical and legal concerns. Data requests should be reviewed with the corresponding author and approved by the Office of Human Research Ethics Committee, Faculty of Medicine, Prince of Songkla University. The datasets analyzed during the current study are available from the corresponding author upon reasonable request.

## Supplementary Materials

The following supporting information can be downloaded at: https://www.mdpi.com/article/10.3390/molecules29030630/s1. Figure S1: Physiochemical properties and characterization of the co-administration of doxorubicin and curcumin within Na^131^I radiolabeled carboxymethyl chitosan (CMCS) nanoparticles (ICED-N) targeted against the epidermal growth factor receptor (EGFR). (A) Z-average diameter (nm). (B) Polydispersity index (PDI). (C) Z-average diameter (nm) and polydispersity index (PDI) of Na^131^I and ICED-N. (D) Zeta potential (mV). Data presented are mean ± standard deviation (*n* = 3). ns: *p* > 0.05, * *p* ≤ 0.05, ** *p* ≤ 0.01, *** *p* ≤ 0.001, **** *p* ≤ 0.0001; Figure S2. Zero-order mathematical modelling of release kinetics of (A) curcumin and (B) doxorubicin loaded within Na^131^I radiolabeled carboxymethyl chitosan (CMCS) nanoparticles (ICED-N) targeted against the epidermal growth factor receptor (EGFR) over a period of 120 h; Figure S3. Korsmeyer–Peppas mathematical modelling of release kinetics of (A) curcumin and (B) doxorubicin loaded within Na^131^I radiolabeled carboxymethyl chitosan (CMCS) nanoparticles (ICED-N) targeted against the epidermal growth factor receptor (EGFR) over a period of 120 h; Table S1. The release exponent value (*n*) of the Korsmeyer–Peppas mathematical modelling of release kinetics mechanism; Table S2. Curcumin and doxorubicin release kinetic and mechanism(s) from ICED-N using the Korsmeyer–Peppas mathematical modelling; Quantification of carboxymethyl chitosan (CMCS) nanoparticle and anti-human EGFR antibody surface coverage.
