# Supplementary material for: Cytotoxicity Enhancement in Osteosarcoma with Multifunctional I-131 Radiotherapeutic Nanoparticles: In Vitro Three-Dimensional Spheroid Model and Release Kinetics Modeling
Source: Molecules. 2024 Jan 29;29(3):630. doi: 10.3390/molecules29030630 (PMC10856476; doi:10.3390/molecules29030630)
Supplement: Supplementary file 1 [file molecules-29-00630-s001.zip › molecules-2802602-supplementary.pdf]

molecules

MDPI

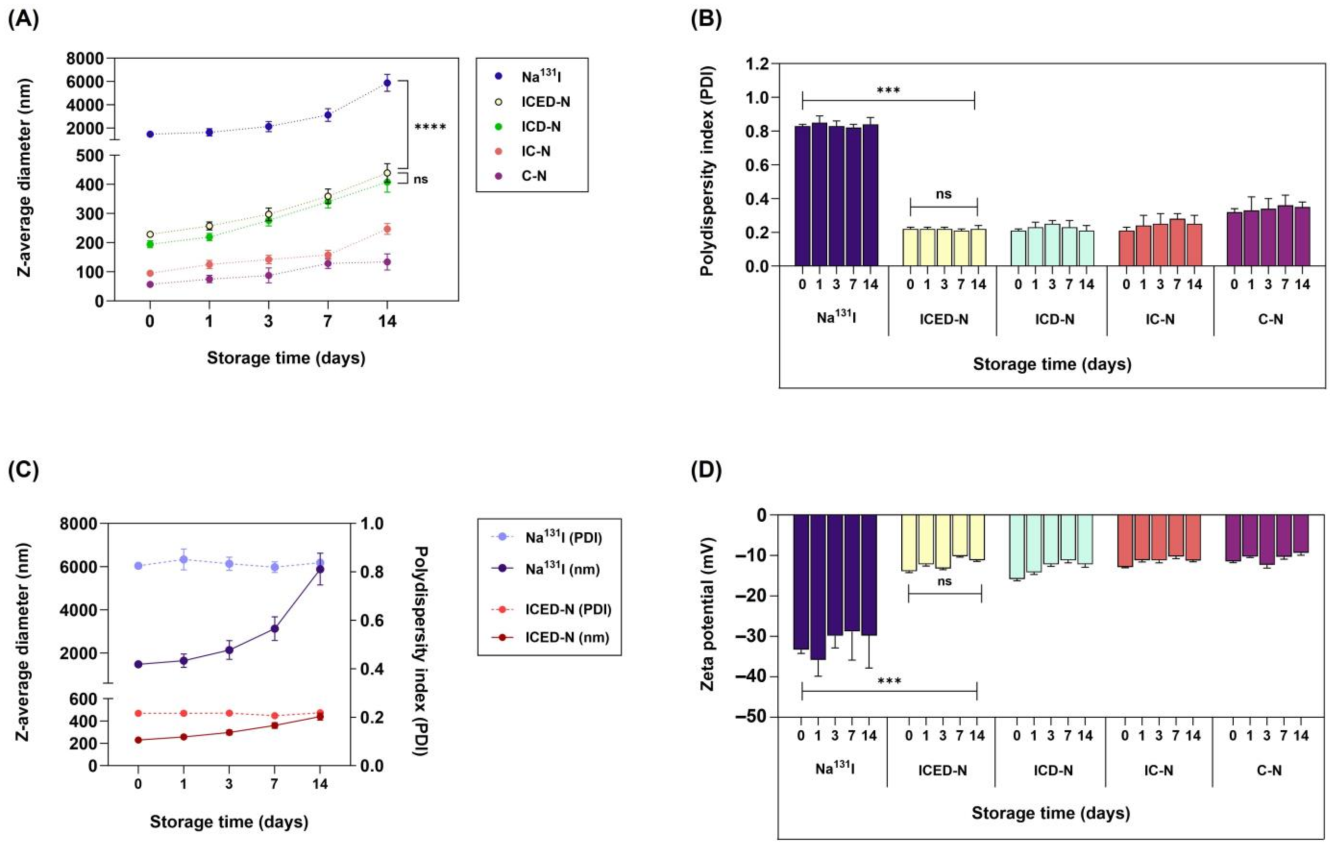

Figure S1. Physiochemical properties and characterization of the co-administration of doxorubicin and curcumin within $Na^{131}I$ radiolabeled carboxymethyl chitosan (CMCS) nanoparticles (ICED-N) targeted against the epidermal growth factor receptor (EGFR). (**A**) Z-average diameter (nm). (**B**) Polydispersity index (PDI). (**C**) Z-average diameter (nm) and polydispersity index (PDI) of $Na^{131}I$ and ICED-N. (**D**) Zeta potential (mV). Data presented by mean ± standard deviation (n = 3). ns: $p > 0.05$, *** $p \leq 0.001$, **** $p \leq 0.0001$.

Quantification of Carboxymethyl Chitosan (CMCS) nanoparticle and Anti-Human EGFR Antibody Surface Coverage

For every 1 mg of CMCS, 4.49×10^{12} molecules of Alexa Fluor 647-labeled anti-human EGFR were introduced onto the surface of nanoparticles. Using Nanoparticle Tracking Analyzer (NTA) (NanoSight, Malvern Panalytical, Malvern, United Kingdom), approximately 3.5×10^{9} particles/1 mg CMCS was measured.

1. For each production run we used 50 mg CMCS contains
   CMCS molecular weight = 543.5 g/mol
   CMCS diameter = 250 nm, radius = 125 nm
   1.1 Surface Area (SA) × mg NP

$$\text{Surface Area} \times \text{ mg NP} = (4\pi r^2)(\text{mg NP}) = (4\pi 125^2)(50 \text{ mg NP})$$

$$= 9.82 \times 10^6 \text{ nm}^2 \times \text{mg NP}$$

Therefore, the Surface Area (SA) × mg CMCS was 9.82×10^6 nm$^2\times$ mg NP.

   1.2 Volume × mg NP

$$\text{Volume} \times \text{mg NP} = \left[\frac{4}{3}\pi r^3\right](\text{mg NP}) = \left[\frac{4}{3}\pi 125^3\right](50 \text{ mg NP})$$

$$= 4.09 \times 10^{8}\ \text{nm}^{3} \times \text{mg NP}$$

Therefore, the volume × mg CMCS was $4.09 \times 10^{8}\ \text{nm}^{3} \times \text{mg NP}$.

1.3 SA:Volume Ratio per mg NP

$$\text{Surface Area: Volume Ratio per mg NP} = \frac{\text{Surface Area}}{(\text{Volume})(\text{mg NP})}$$

$$= \frac{(4\pi 125^{2})\text{nm}^{2}}{\left[\frac{4}{3}\pi 125^{3}\right]\text{nm}^{3}\ (50\ \text{mg NP})}$$

$$= \frac{1.96\times 10^{5}}{(8.18\times 10^{6})(50)}$$

$$= 4.79 \times 10^{-4}\ \frac{\text{nm}^{2}}{\text{nm}^{3}\times \text{mg NP}}$$

Therefore, the SA:Volume ratio per mg CMCS was $= 4.79 \times 10^{-4}\ \frac{\text{nm}^{2}}{\text{nm}^{3}\times\text{mg NP}}$

1.4 Number of molecules × mg NP

$$\text{Number of molecules} \times \text{mg NP} = \left[\frac{50 \times 10^{-3}\text{g}}{543.5\ \text{g/mol}}\right] \times (6.02 \times 10^{23}\ \text{mol}^{-1})$$

$$= 5.54 \times 10^{19}\ \text{molecules}$$

Therefore, the number of molecules × mg CMCS was $= 5.54 \times 10^{19}$ molecules.

1.5 Particle number of CMCS: approximately 3.5×10^{9} particles/1 mg CMCS

$$= \frac{3.5 \times 10^{9}\ \text{particles}}{1\ \text{mg CMCS}} \times 50\ \text{mg CMCS} = 1.75 \times 10^{11}\ \text{particles}$$

Therefore, the particle number of CMCS was 1.75×10^{11} particles.

2. To functionalized Alexa Fluor 647-labeled anti-human EGFR to CMCS nanoparticles
Alexa Fluor 647-labeled anti-human EGFR: MW 134,000 g/mol

$$\text{Anti-EGFR 1 μg} = \frac{(1 \times 10^{-6}\ \text{g})(1\ \text{mol})}{134{,}000\ \text{g}} = 7.46 \times 10^{-12}\ \text{mol}$$

$$\text{Number of anti-EGFR molecules} = (7.46 \times 10^{-12}\ \text{mol})(6.02 \times 10^{23})$$

$$= 4.49 \times 10^{12}\ \text{molecules}$$

Therefore, the number of anti-EGFR molecules was 4.49×10^{12}.

Zero-Order Mathematical Modelling of Release Kinetics of Curcumin and Doxorubicin-Loaded ICED-N

An indicator of the degree to which the regression predictions match the actual data points, the R^2 coefficient of determination, is a statistical metric. If R^2 equals 1, then the regression predictions match the data precisely. The ideal drug release mathematical model for curcumin and doxorubicin experimental data was selected based on its capacity to produce the highest correlation coefficient (R^2) with the release data.

The zero-order model correlation coefficient was 0.8755 for curcumin (Figure S2A) and 0.8601 for doxorubicin (Figure S2B), based on the decreased correlation coefficient value, it can be inferred that the release of curcumin and doxorubicin fails to follow zero-order release kinetics.

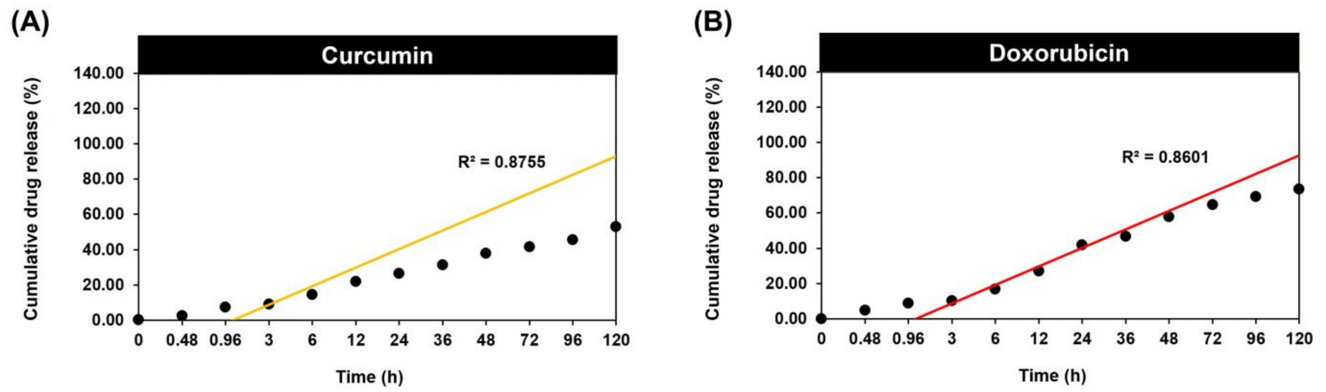

Figure S2. Zero-order mathematical modelling of release kinetics of (**A**) curcumin and (**B**) doxorubicin loaded within $Na^{131}I$ radiolabeled carboxymethyl chitosan (CMCS) nanoparticles (ICED-N) targeted against the epidermal growth factor receptor (EGFR) over a period of 120 h.

Korsmeyer–Peppas Mathematical Modelling of Release Kinetics of Curcumin and Doxorubicin-Loaded ICED-N

The results of the Korsmeyer–Peppas mathematical model determined a curcumin R^2 value of 0.9184 (Figure S3A) and a doxorubicin R^2 value of 0.83569 (Figure S3B), based on the lower correlation coefficient values, it is suggested that the release of curcumin and doxorubicin does not conform to Korsmeyer–Peppas model.

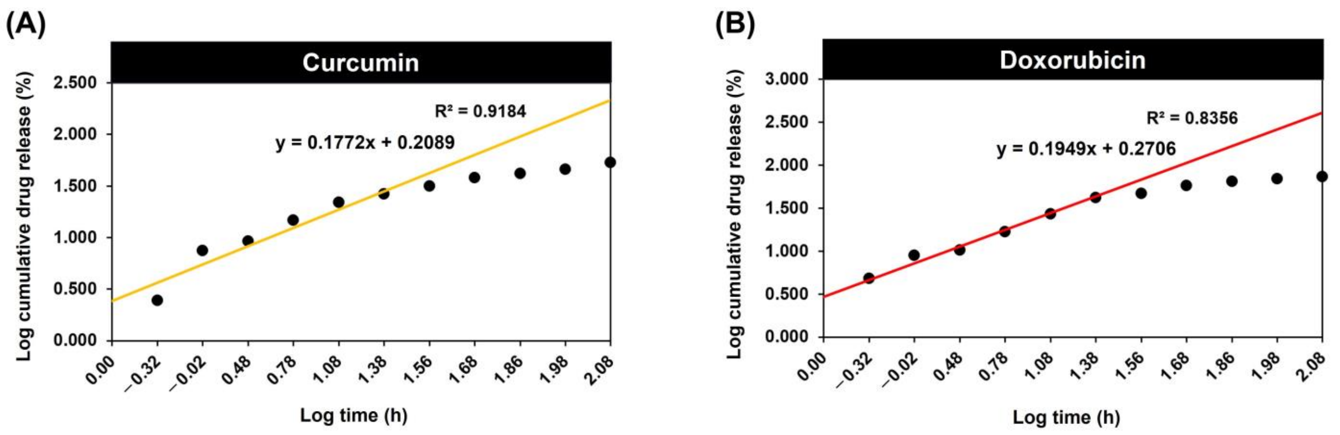

Figure S3. Korsmeyer–Peppas mathematical modelling of release kinetics of (**A**) curcumin and (**B**) doxorubicin loaded within $Na^{131}I$ radiolabeled carboxymethyl chitosan (CMCS) nanoparticles (ICED-N) targeted against the epidermal growth factor receptor (EGFR) over a period of 120 h.

The release exponent (n) and corresponding drug delivery mechanisms are shown in Table S1. As can be seen, the curcumin release exponent (n) value is 0.1772, which fits the range $0 < n < 0.5$, indicating the release mechanism is Hindered Fickian, which results in impeded drug release. Similarly, the doxorubicin release exponent (n) value of 0.1949 also fits within this range, indicating impeded drug release. The Korsmeyer–Peppas mathematical model calculation (Table S2) resulted in curcumin having a release exponent (n) of 0.1772 and doxorubicin having a release exponent of 0.1949. This suggests that the release mechanisms of curcumin and doxorubicin follow Hindered Fickian Diffusion (Quasi-Fickian Diffusion). The early rapid release of doxorubicin and curcumin (Figure 3) within the first 12 h is a result of their dissolution from the surface of the CMCS nanoparticles.

Table S1. The release exponent value (n) of the Korsmeyer–Peppas mathematical modelling of release kinetics mechanism.

Release Exponent Value (n)	Release Mechanism	Release Kinetic and Mechanism(s)
$0 < n < 0.5$	Hindered Fickian (Quasi-Fickian) diffusion	The diffusive transport process where hindrances or barriers within the CMCS matrix impede the smooth diffusion of curcumin and doxorubicin. This situation involves the presence of obstacles that significantly decelerate the overall diffusion process.
$n = 0.5$	Fickian diffusion controlled release	The release of curcumin and doxorubicin is predominantly controlled by the diffusion of the drug through the CMCS matrix.
$0.5 < n < 1$	Non-Fickian or Anomalous Transport (Case II)	The release is not purely Fickian and might involve swelling, erosion, or relaxation processes in addition to the diffusion.
$n = 1$	Zero-order release kinetics	The release is predominantly controlled by polymer relaxation and erosion rather than diffusion.
$n > 1$	Super Case II Transport	The release is faster than predicted by Case II transport, which usually occurs where significant modifications in the CMCS matrix take place.

Table S2. Curcumin and doxorubicin release kinetic and mechanism(s) from ICED-N using the Korsmeyer–Peppas mathematical modelling

Drug	Equation	Release Exponent Value (n)	Release Mechanism
Curcumin	$y = 0.1772x + 0.2089$	0.1772	Hindered Fickian (Quasi-Fickian) diffusion
Doxorubicin	$y = 0.1949x + 0.2706$	0.1949	Hindered Fickian (Quasi-Fickian) diffusion
